# Solar energy policies in southeast Asia towards low carbon emission: A review

**DOI:** 10.1016/j.heliyon.2023.e14294

**Published:** 2023-03-11

**Authors:** Logeswaran Govindarajan, Mohd Faizal Bin Mohideen Batcha, Mohammad Kamil Bin Abdullah

**Affiliations:** a Faculty of Mechanical and Manufacturing Engineering, University Tun Hussein Onn Malaysia, 86400, Parit Raja, Batu Pahat, Johor, Malaysia; b Center for Energy and Industrial Environment Studies (CEIES), University Tun Hussein Onn Malaysia, 86400, Parit Raja, Batu Pahat, Johor, Malaysia

**Keywords:** Net energy metering, Solar energy policy, Renewable energy, Carbon emission

## Abstract

Globally, countries are developing policies and encouraging the implementation of sustainable energy resources to reduce the harmful effects of fossil fuels on the environment and energy-related CO_2_ emissions. In 2019, global energy-related CO_2_ emissions increased by 1.8% to a new high of 33.3 Gt CO_2_, owing to the increasing energy consumption. The CO₂ emissions are significantly increasing due to continuing increase of Southeast Asian countries. Energy utilization contributes to CO_2_ emissions on earth because the energy sector produces 32,553.48 MtCO_2_ of CO_2,_ or about 73% of total CO₂ emissions (WRI, 2019). The power sector alone accounted for approximately two-thirds of the emissions rise, indirectly warming the climate system, earth's temperature, and sea level. As a result, several governments have enacted policies to increase solar energy's share of the energy mix to minimize dependence on fossil fuels and environmental devastation. Therefore, this review paper presents a survey of solar energy policies implemented in Southeast Asian countries, specifically Malaysia, and assesses effective existing solar energy strategies in developed countries. Moreover, the implementation of Net Energy Metering needed for the advancing and widespread use of renewable energy technologies is also reviewed. Malaysia's existing solar energy policies have also been assessed and compared to the selected high-income nations. Lastly, limitations and key challenges of implementing large-scale applications of net energy metering policies are also presented.

## Highlights

• The importance of implementing sustainable and clean energy technologies due to global energy-related CO_2_ emissions is presented.
• Total final energy consumption (TFEC), utilization of net final energy by sectors and primary energy supply for each Southeast Asia country has been explained.
• The transition of solar energy polices in Southeast Asia countries is discussed.
• The detailed structure of Net Energy Metering (NEM) policies, including eligible sectors, capacity, renewable energy target and tariff for surplus energy in Southeast Asia countries, is addressed.
• Case study on addressing achievement of implementation of Net Energy Metering 2.0 interms of financial benefits to consumers described in this section.

## Graphical abstract

Graphical Implementation of RE with Grid

**Figure:**
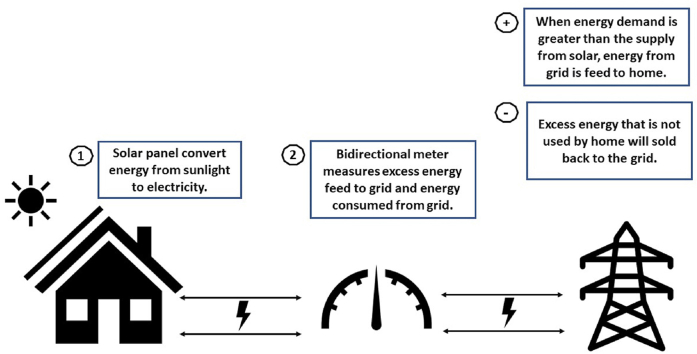

## List of symbols and abbreviations

ASEAN: Association of Southeast Asian Nations
AEDP: Alternative Energy Development Plan
AMEM: ASEAN Ministers on Energy Meeting
APAEC: Action Plan on Energy Cooperation
BAU: Business as usual
CO2: Carbon Dioxide
CAREC: Central Asia Regional Economic Cooperation
COP: Conference of the Parties
DUs: Distribution Utilities
DIS: Distribution Impact Study
DL: Distribution Licensee
EC: Energy Commission
EPPO: Energy Policy and Planning Office
ERC: Energy Regulation Commission
EVN: Electricity Vietnam
FiT: Feed in Tariffs
GHG: Green House Gases
GWh: Gigawatt hour
IRENA: International Renewable Energy Agency
IUPTLU: Izin Usaha Penyediaan Tenaga Listrik untuk Kepentingan Umum
kWh: Kilowatt-hour
KETSA: Ministry of Energy and Natural Resources
MW: Megawatt
MEA: Metropolitan Electricity Authority
MEMR: Minister of Energy and Mineral Resources
MD: Maximum Demand
NDC: Nationally Determined Contribution
NZE: Net Zero Emission
NRC: National Reform Council
PEA: Provincial Electricity Authority
PPA: Power Purchase Agreement
PLN: Perusahaan Listrik Negara
PV: Photovoltaics
RE: Renewable Energy
RUPTL: Electricity Business Plan
REC: Renewable Energy Certificate
RPS: Renewable Portfolio Standard
SMP: System Marginal Price
STC: Standard Test Condition
SEDA: Sustainable Energy Development Authority
TFEC: Total final energy consumption
TBH: Thai Baht
TNB: Tenaga Nasional Berhad
UNFCCC: United Nations Framework Convention on Climate Change
UTHM: University Tun Hussein Onn Malaysia
VEPG: Vietnam Energy Partnership Group
VND: Vietnamese dong
WRI: World Resources Institute

## 1 Introduction and research motivation

As the world responds to calls for ‘more sustainable energy’ and ‘less carbon,’ Renewable Energy (RE) has emerged as one of the fastest-growing energy generation sources [1]. Until 2040, RE is predicted to remain the major source of energy. Any form of total energy obtained from renewable sources, including solar energy, biomass, biogas, mini-hydro, and geothermal power, is referred to as RE. Due to the growing magnitude of climate change, the need to mitigate the adverse consequences of fossil fuels, and widespread disquiet among global communities, many countries have modified their energy strategies to increase the percentage of RE [1,2]. 179 nations had submitted RE development goals, with 57 proposing RE electricity share targets of 100% by 2017 [3,4]. For instance, the European Union (EU) has mandated that by 2020, its members meet 20% of their energy needs with renewable energy [5]. Moreover, when they endorsed the Paris Agreement, nations agreed to lower CO_2_ and other greenhouse gas emissions and respond to the consequences of climate change [6,7]. The pollutant sources are generally increasing with rapid development and industrial activity. Which ultimately poses a challenging task to the government to maintain ecological integrity while increasing the community's standard of living. Countries may reduce one significant source of the problem: energy-related CO_2_ emissions, climate system warming, earth's temperature, and sea level rising by enlarging renewable energy [8].

Energy policy, in general, is a major driving force behind the growth of renewable energy. Energy generation accounts for more than a third of all greenhouse gas emissions, making it the economy's most important sector. Governments across the country have created various policies related to green technology to drive green technology in their respective countries for environmentally friendly (hybrid) vehicles to change society's mindset towards environmental sustainability and positive development to improve public awareness.

Moreover, Modern societies depend on electrical energy in various sectors, such as health, security, economy, and communication systems. The electricity demands keep increasing, and its generation uses hydrocarbon fuels, mainly coal leading to CO2 emission and the greenhouse effect. Due to these problems, efforts to increase Renewable Energy (RE) participation in generating electricity rapidly worldwide. Malaysia, for example, had targeted increasing the RE share to 31% or 12.9 GW in 2025 and 40% or 18.0 GW in 2035 [9]. Every nation concerned about climate change should effectively make a green power policy a primary focus [10]. Because the combustion of fossil fuels generates most of the energy, a green power policy must encourage the use of environmentally friendly substitutes like solar, wind, and hydroelectric power while also devising ways to discourage the consumption of coal, oil, and gas. Thus, countries worldwide are committed to creating a green culture and preserving the environment to reduce carbon emissions by adopting an efficient energy policy ahead of 2030 [9,11]. Generally, this energy policy is important in adapting the energy management initiatives optimally, adopting energy-saving to reduce carbon emissions, and applying green technology to improve energy efficiency by the citizen. Therefore, this paper analyzes the long-term goals and energy policies to cultivate and implement research toward green technology. Moreover, the detailed structure of NEM policies in southeast Asian countries is also reviewed. The main objectives of this review paper are:

1. To review the importance of implementing Renewable Energy Technologies
2. To analyze net energy utilization by sectors in each Southeast Asia country.
3. The transition of solar energy policies in Southeast Asia countries is discussed.
4. To review the advantages, challenges, and key limitations of implementing Net Energy Metering (NEM) policies and their tariff for surplus energy in Southeast Asian countries.

## 2 Literature review

According to the reports of the International Renewable Energy Agency (IRENA) 2021 [7,12], the integration of RE sources and technologies accounts for 37% of total installed capacity globally. Therefore, numerous investigations have compiled a list of RE policies and their current state of development in different nations. The statistics of implementing RE share in total energy generation are presented in detail in the literature [13,14]. Boie (2016) split government policies into two categories: production incentives and investment incentives. He investigated the motivational effect of renewable energy incentive systems [15]. Maslin and Scott (2011) predicted future RE expansion on a global scale using a variety of possibilities. They found that environmental constraints would restrict RE growth and that technology developments would also decide the magnitude of RE and its ability to connect with traditional energy systems [16]. Moreover, Malaysia aims to transit its RE energy share from the existing 23% RE to 40 by 2035 [12].

According to the world bank report for South Asian developing countries like Pakistan, it is mentioned that utilizing 0.071% of the total country area, this country has tremendous potential to generate wind and solar power. Hence, more than 30% RE is planned to add to the system by 2030 to improve and strengthen the power quality [17]. Such RE integration is capable of mitigating CO2 emissions. Moreover, in a country report of Central Asia Regional Economic Cooperation (CAREC energy outlook), it is emphasized to take up strategic plans in implementing RE to mitigate both emissions and supply risks to provide sustainability and security of the regional energy sector [18]. Similarly, a study conducted for Bangladesh from 1972 to 2006 is discussed in Refs. [14,19], emphasizing implementing RE to meet the energy demand. Moreover, the studies are presented in Refs. [20,21] on group method data handling to mitigate CO2 emissions by modelling various energy systems of G8 countries. A comparative study based on different study periods, variables, and methodological applications was conducted in Refs. [22,23] to ensure the energy policy implications by focusing on RE transition to ensure economic growth and tackle CO2 issues. Therefore the policies seem to have an apparent divergence either in target or implementation.

Southeast Asian nations actively aim to boost the proportion of renewable energy sources to mitigate their reliance on fossil fuels and document the policies for implementing renewable generations. In Ref. [24], the authors have discussed government policy in detail on RE, which is the key accelerator of RE development in the energy sector. Moreover, many national energy councils of ASEAN are regularizing the mechanism and guidelines on RE standards and net metering policies [[25], [26], [27]]. These policies include net energy metering (NEM), feed-in tariffs (FiT), pricing laws, the renewable portfolio standard (RPS), and tax credits. As a result, the most popular policy is NEM, despite many disagreements over its effectiveness, making it difficult to choose the best one. The choice of RE policies for execution will depend on the circumstances and development goal of the country. Nevertheless, the impact of these restrictions varied widely depending on the stage of development of the RE sector. Therefore, this paper will give an in-depth review and comparison of NEM legislation between Malaysia and several Southeast Asia nations, namely Thailand, Vietnam, the Philippines, and Indonesia. Apart from that, net-metering advancements and challenges for future RE targets in the Asian region are also addressed.

## 3 Energy policies in Southeast Asian countries

### 3.1 Thailand

**Fig. 1:**
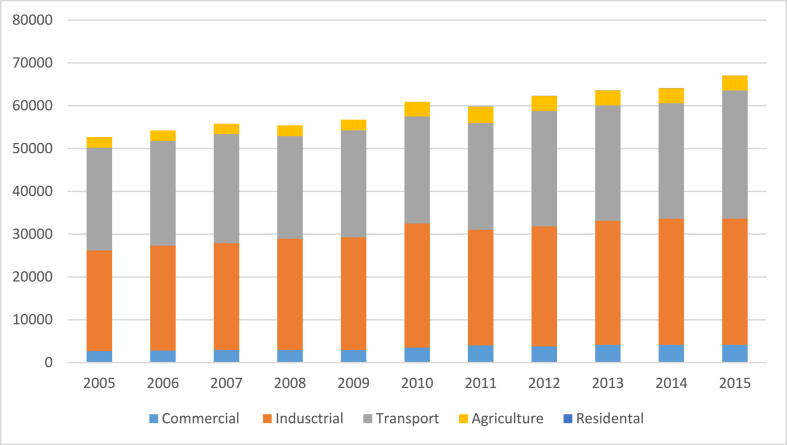
Thailand's energy usage, clustered by economic sector, from 2005 to 2015 [28].

**Fig. 2:**
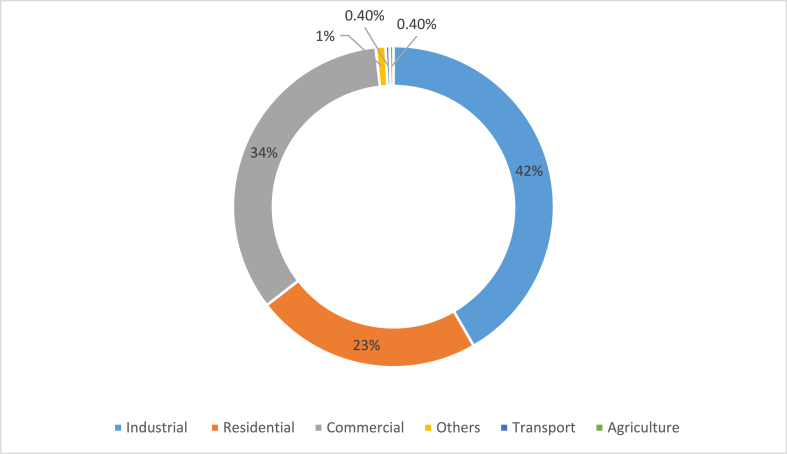
Utilization of electricity by the economic sector [28].

In the past 10 years, Thailand's total final energy consumption (TFEC) has constantly been expanding, as shown in Fig. 1. Three-quarters of the total was consumed by the industrial and transportation sectors [28,29]. The industrial sector (76,910 GWh or 42%), the commercial sector (61,450 GWh or 34%), and the residential sector (41,450 GWh or 22%) were the largest energy users throughout the year, as shown in Fig. 2 [28,30].

**Fig. 3:**
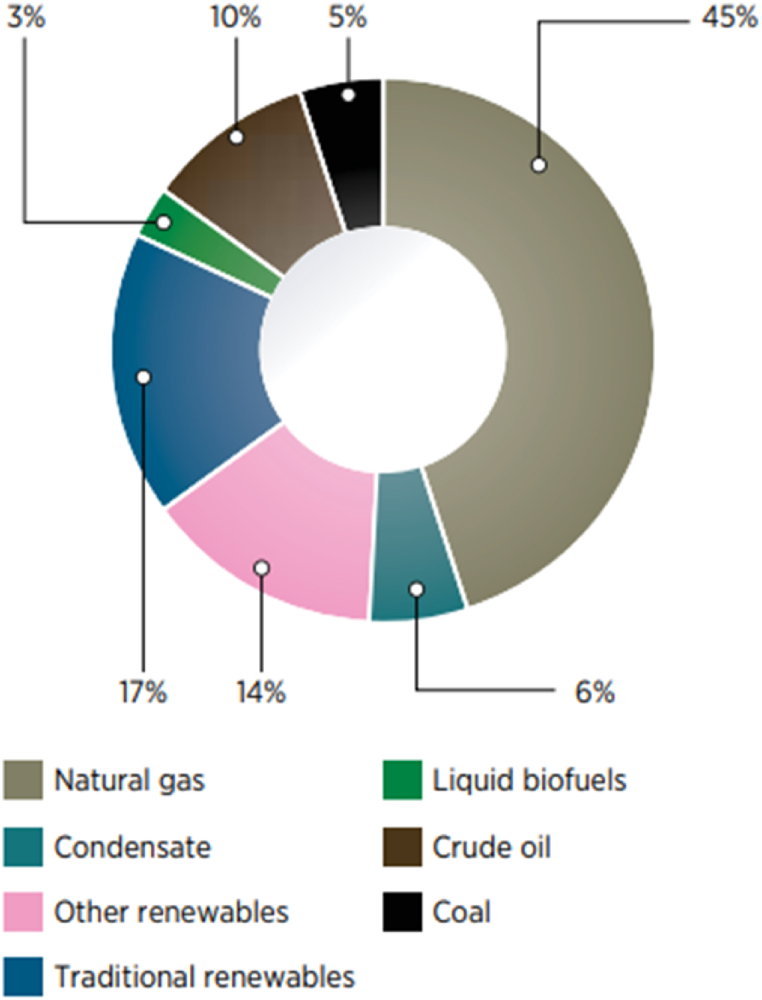
Thailand's entire primary energy production [28].

As illustrated in Fig. 3, natural gas, condensate, and crude oil accounted for 61% of the principal energy supplies from indigenous traditional sources in 2015. Regarding final energy consumption, oil-derived product groups and fuel gas accounted for around 72% of Thailand's total final energy consumption [3,28]. As a result, the imported energy or domestic lignite coal usage would rise to compensate for the loss of natural gas, condensate, and crude oil. The speedily rising energy use has now raised worries over supply difficulties. Moreover, The large consumption of fossil energy resources has resulted in heavy environmental consequences like ozone layer degradation, climate change, and global warming. To mitigate the negative environmental effects, Thailand's government has emphasized renewable energy production over the last few decades [31,32].

**Fig. 4:**
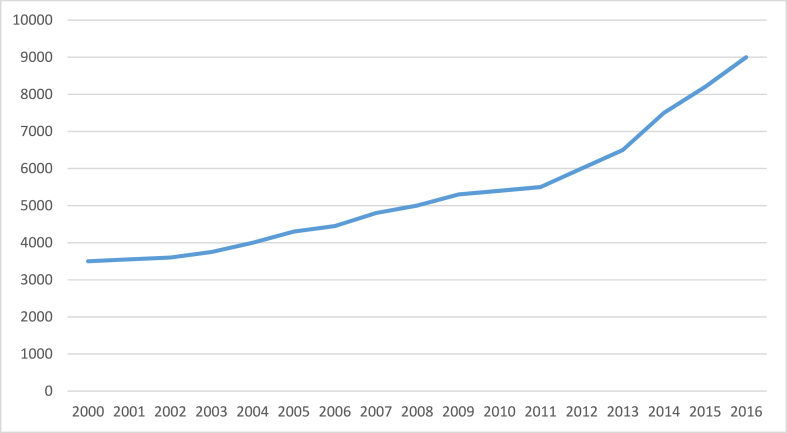
Overall renewable electricity generation capacity in Thailand, 2000–2016 [28].

Thailand had acquired a respectable percentage of renewable energy in primary energy output by 2015, especially by promoting solar PV systems, as shown in Fig. 3 [30]. In 2015, the annual growth rate of modern renewable energy was 11.69%, more than four times the annual growth rate of the total primary energy supply. In 2015, almost 64% of the total renewable energy usage (10,360 ktoe) was utilized for thermal generation, 16% for generating electricity, and roughly 20% for biofuels [[33], [34], [35]]. In the electricity sector, installed renewable energy-producing capacity has more than tripled in the last decade, with a stronger ramp-up since 2012, as seen in Fig. 4.

Thailand's government has acknowledged the need for alternative energy sources, particularly renewable energy sources, and has recognized the need to implement programs to encourage renewable energy research and implementation [36]. The Ministry of Energy has therefore prepared the "National Energy Plan" to serve as a framework for guidelines and policy values for the future energy development of Thailand. In preparing this new national energy plan, the existing 5 national energy plans will likely comprise the country's power development plan, renewable and alternative energy development plans, energy conservation plan, natural gas management plan, and fuel management plan. Ministry of Energy, The Energy Policy and Planning Office or EPPO is in the process of preparing the details of the National Energy Plan that is consistent with the approach towards the target to reduce carbon emissions to zero (Carbon Neutrality) within “2065–2070”, which will affect the direction of important energy development.

According to the Alternative Energy Development Plan (AEDP 2015) [28], Thailand has defined a target of using renewable energy sources for 30% of ultimate energy consumption by 2036. Renewable energy has provided electricity for 20.11% of total energy consumption, corresponding to a cumulative installed capacity of 19,684.4 MW (comprising hydropower capacity). By 2036, the goal for solar PV will be set at 6,000 MW of installed capacity. The incentive program is a strategy for promoting the growth of solar PV in Thailand [37]. Two incentive schemes have been in effect since 2007 to promote the expansion of solar PV technology. In 2007, the first incentive program, the Adder program, was introduced. The Adder program includes a standard tariff (ranging from 3.06 to 3.17 Thai Baht Per kWh) and a premium [38]. From 2007 to 2010, the premium for solar PV was fixed at 8 Thai Baht per kWh. FiTs (feed-in-tariff) are the second incentive program [39]. The community ground-mounted solar program will be the second FiTs program (1 Tambon, 1 MW). This scheme included a three-step degression, with rates of 9.75 Thai Baht/kWh for years 1–3, 6.50 Thai Baht/kWh for years 4–10, and 4.50 Thai Baht/kWh for years 11–25 [38]. For this FiTs plan, the government has set aside an 800 MW quota; the systems must be deployed by December 2014. Rooftop solar PV installations were subsequently legalized by the National Reform Council (NRC) in January 2015, which replaced the "adder" scheme with the FiT plan, allowing all houses to install and connect them to the electricity grid [40]. After a calm spell in the renewable energy market in 2014, the regulation attempts to rekindle investment in renewable energy projects in Thailand [41]. The rooftop solar PV net metering pilot project demonstrates policymakers' desire to shift away from FiTs and toward a market-driven by self-consumption. ERC formally announced the initiative on August 11, 2016. By January 31, 2017, the systems must be installed. The concept divides 50 MW between the Provincial Electricity Authority (PEA) and the Metropolitan Electricity Authority (MEA) for distribution throughout all provinces [42].

The 10-year net metering rate will be THB1.68/kWh ($0.052), significantly less than the current home electricity pricing of THB3.80/kWh [43]. Solar system owners will also have to pay a THB8, 500 grid connection fee. The new metering tariff, which is substantially lower than the current one, will be set for 10 years as part of the recently updated power development plan, which aims at having 35% of total power supplied by renewable sources by 2037 as the current clean energy accounts for just 10% of the supply [43,44]. The target for solar energy by 2036 is set at 6 GW, 50% of which was achieved in 2017. Thailand's present solar capacity of 3.3 GW represents more than 60% of the Association of Southeast Asian Nations (ASEAN) total installed capacity [45]. Recent reports speculate that the target could be raised to 17 GW with current success in ongoing net energy metering. Overall, it could be said the framework energy policies aim to reduce net-zero carbon drive energy policy to support the trend of economic transition to a low carbon economy. Energy policies under the national energy plan will be the framework and direction of the plan to move towards more clean energy and to show the position and preparation of the adjustment to support the line shifting the economy to a low-carbon economy (Neutral-carbon economy), increasing Thailand's competitiveness and the opportunity to attract investment from foreign investors in which people participated from the beginning in the preparation of the plan to jointly determine the direction of Thailand's energy policy in the future.

### 3.2 Vietnam

Energy serves a critical function in the country's socio-economic growth; as a result, the Party and State have focused their development policies in subsequent years on enhancing the standard of energy consumption. In general, sustainable development of the energy sector and national energy security is a key elements in sustainable development and enhancement of the country's competitiveness. Over the years, as an industry management agency, the Ministry of Industry and Trade has implemented many specific solutions, from policy consulting and financial, technical, and technological support to promoting information and propaganda. Consequently, from 2006 to 2015, Vietnam saved about 16 million tons of oil, equivalent to about 103.7 billion kWh of electricity.

Vietnam's ultimate energy consumption mix in 2017 was 62.5 million tonnes of oil (Mtoe), up 5.5% yearly and 4.3 times higher than the 1990 level of 17.4 Mtoe [46,47]. Transportation had the highest annual growth rate (8.85%), trailed by industrial (7.76%) and residential/commercial (1.4%). There will be a 12.15% yearly increase in non-energy use. Between 1990 and 2050, Fig. 5 illustrates the ultimate energy utilization by sectors [46,48].

**Fig. 5:**
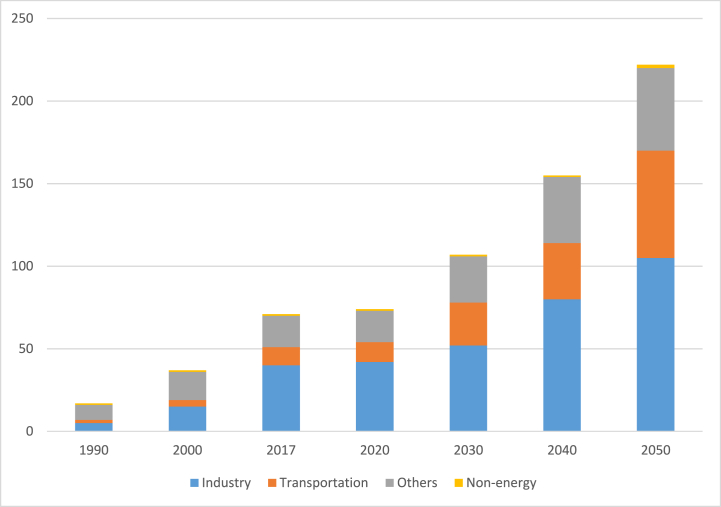
Final Energy Consumption by sector, BAU (1990–2050) [50].

**Fig. 6:**
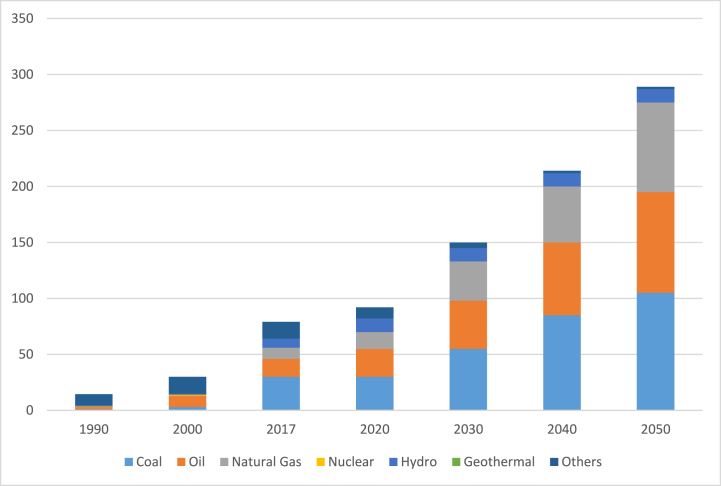
Primary energy supply, BAU (1990–2050) [50].

From 17.9 Mtoe in 1990 to 78.9 Mtoe in 2017, Vietnam's main energy supply increased quicker than final energy usage, increasing at a rate of 5.7% per year, or 4.4 times, from 17.9 Mtoe in 1990 to 78.9 Mtoe in 2017. Natural gas, hydro, coal, and oil were the fastest-growing primary energy sources [46,49]. Natural gas usage increased by 33.8% per year between 1990 and 2017, whereas hydro, coal, and oil consumption increased by 10.9%, 10.2%, and 7.6% per year. In a Business-as-Usual (BAU) scenario, Vietnam's total energy production is predicted to surge by 4.1%, or 3.7 times, from 78.9 Mtoe in 2017 to 293.1 Mtoe in 2050 [50]. Natural gas is predicted to expand at the quickest pace, growing at a 7.5% annual average rate between 2017 and 2050, followed by oil (4.6%), coal (3.9%), and hydro (1.2%), while other fuels (mainly biomass) would decline at a 6.1% annual rate [49]. Fig. 6 depicts the primary energy supply by source from 1990 to 2050 [46,49].

To fulfill this growing energy need, the Vietnamese government notably declared a wave of different policies and programs primarily directed at expanding the proportion of renewable energy in energy production, especially solar PV systems, as well as boosting energy efficacy to close the gap between energy supply and usage and decrease greenhouse gases GHG emissions [46,51,52].

In Decision No. 280/QD-TTg, the Prime Minister urged the People's Committees of provinces and centrally-run cities to develop and approve the plan to implement the program in their localities, clearly stating the roadmap and objectives, which are to consume energy economically and efficiently according to its competence; the arrangement of funding for implementation, inspection, supervision and assessment of quality, progress, etc. to implement the economical and efficient use of energy in the locality. Research and promulgate mechanisms and policies to give special incentives and rewards to organizations and individuals with achievements in economical and effective energy utilization in the locality. The government also emphasizes organizing and coordinating programs to propagate the locality's economic and effective energy utilization. Direct functional units to strengthen inspection and examination and urge the law's implementation on economical and effective energy utilization.

According to Mr. Phuong Hoang Kim - Director of the Department of Energy Saving and Sustainable Development (Ministry of Industry and Trade), to organize to achieve the goal of saving 8–10% of total energy consumption compared to the development scenario. Usually, by 2030, the participation of local authorities at all levels will play a significant and decisive role. To achieve this goal, Mr. Phuong Hoang Kim said, it is necessary to implement many solutions in parallel. Accordingly, to improve the behaviour of using electricity/energy economically and efficiently, it is necessary to promote propaganda, raise awareness, and improve the behaviour of using electricity/energy for each specific target group with many different forms, such as workers, residential communities, tourists, students with the participation of many mass organizations and social organizations.

Consequently, Vietnam has established itself as the frontrunner in the ASEAN area in terms of adopting renewable energy sources, for instance, solar and wind power [53]. In 2019, the nation overtook Thailand with the highest combined solar and wind power installed potential. It is anticipated that Vietnam's total solar photovoltaic (PV) capacity will have acquired roughly 16,500 MW (MW) by the end of the year 2020 [12]. Which greatly exceeds the initial 2020 objective of 850 MW (Government of Vietnam, 2016), and it is possibly moving near to the provisional aim of 18,600 MW of installed solar power capacity by 2030 set out in the draft version of Vietnam's Power Development Plan 8 (Government of Vietnam, 2016). A great accomplishment was achieved in Vietnam in 2019 and 2020 by installing more than 100,000 rooftop solar PV systems [53]. Despite the fact that 2020 was bleak, with signs of ASEAN energy progress being pushed back due to the COVID-19 outbreak, Vietnam achieved new records for solar capacity. Rooftop solar systems contributed 9.3 GW (GW) to the power network in the country. By December 2020, Vietnam will have increased rooftop solar by eightfold, from only 378 MW (MW) in 2019. With Vietnam appearing as the area's new RE tiger, eclipsing Thailand as the region's earlier pioneer, the region is projected to move closer to its 2025 RE goal.

The Vietnamese government delivered Decision No. 11/2017/QD-TTg on encouraging the growth of solar power, which plays a major role in the country's renewable energy development [54]. This Decision No. 11/2017/Q-TTg is the fundamental legal document mandating rooftop projects to use two-way measuring meters to achieve the "net-metering" system [55]. In terms of net metering, the Ministry of Trade and Industry in Vietnam will establish annual purchasing and offering prices for rooftop grid-connected PV systems on the basis of the VND/$ exchange rate. Excess electricity compensation is 9.35 US cents per kWh (2,086 VND) and is revised annually based on the VND-USD currency rate [56]. If the quantity of electricity produced during a payment cycle exceeds the amount utilized, the excess will be carried over to the next payment cycle. The excess electricity will be transferred to the local power organization Electricity Vietnam (EVN) at the prevailing Feed-in-tariff price at the end of the year or upon the expiration of the Power Purchase Agreement ("PPA").

NEM was utilized to coordinate the initial phases of solar development in Vietnam. NEM, income, and land leasing payment concessions are part of the Vietnamese policy system. The government's aim to secure an adequate local electricity supply to meet rising power urges and public requirements for local environmental integrity are underlying motivations [57]. Furthermore, there has been an increase in public support for government policies and initiatives that have enabled an upsurge in the supply of energy while simultaneously reducing the rate at which prices have risen. The emergence of the climate policy's lobbying power has aided in developing and implementing low-carbon measures such as NEM. NEM has elicited strong responses from enterprises and has facilitated the industry's rapid development, particularly in the case of solar [44]. The instance of Vietnam exemplifies how the government, industry, and the general people may collaborate to achieve a greener growth model.

Moreover, Vietnam also continually prioritizes clean energy development by working hard to realize the commitment made by Prime Minister Pham Minh Chinh at the Leaders' Summit within the framework of the 26th United Nations Conference on Climate Change (COP26) toward net-zero emissions reduction by 2050. To achieve this goal, the Ministry of Industry and Trade has developed a plan to revise the "Draft Power Master Plan VIII" to reduce coal power sources sharply and emphasize developing gas power, especially LNG gas power. This energy development is to ensure energy security and increase the ability to absorb electricity using renewable energy will be strongly developed on a large scale, at the same time, promote economic and effective utilization of energy.

Minister of Industry and Trade Nguyen Hong Dien said: “In the Draft Power Plan VIII, the framework of power sources has been calculated with the proportion of renewable energy (mainly wind power) increasing very high. The overall capacity of renewable energy supply electricity sources will reach 38 GW (GW) in 2030, contributing 24%. Hydropower will not be included in this estimate. We know that developed countries like the US are currently only about 14–15%. We raised it to 24–25%; this is very revolutionary. By 2045, both wind power and solar power will thrive. The total capacity of renewable energy sources (except hydroelectricity) will reach about 56 GW, accounting for 45% of the power source structure.

At the 4th Summit of the Vietnam Energy Partnership Group (VEPG) at the end of January 2022 in Hanoi, Deputy Minister of Industry and Trade Nguyen Hoang An emphasized: The Vietnamese Government has stepped up and is determined to implement the transition. Restructuring the energy sector to maximize domestic resources while increasing coordination and assistance from development partners.

According to Deputy Minister Dang Hoang An, Vietnam has several potentials and benefits for developing green and clean energy, including the advantages of natural conditions and the government's initiative and long-term perspective.

Energy and electricity sectors were restructured in tandem with the promotion of reshaping and enhancing the efficacy of state-owned enterprises and the continued equitization and divestment of state capital in enterprises without prioritizing holding, constructing, and progressively establishing a challenging electricity market at all levels in accordance with the approved roadmap. In addition, Vietnam is promoting the third phase of the National Program on Economic and Efficient Energy Use., phase 3.

Aside from the positive reasons, Vietnam must also introduce solutions to address the obstacles and problems of maintaining energy security while limiting the environmental effects of power generation. High-speed load development, in particular, imposes a strain on the energy industry's network, necessitating huge investments in public debt and adverse equitization processes.

As a result, in the development orientation of Vietnam's energy sector, a long-term, durable, and appropriate energy industry expansion strategy is critical.

With increasing solar in the southern region of Vietnam, additional linkages with its ASEAN neighbour are more plausible. Vietnam has become a new RE trading alternative for ASEAN due to the existing solar boom. Vietnam's renewable energy surge would probably spark new multilateral trading under the ASEAN Power Grid in the near future [58].

### 3.3 The Philippines

**Fig. 7:**
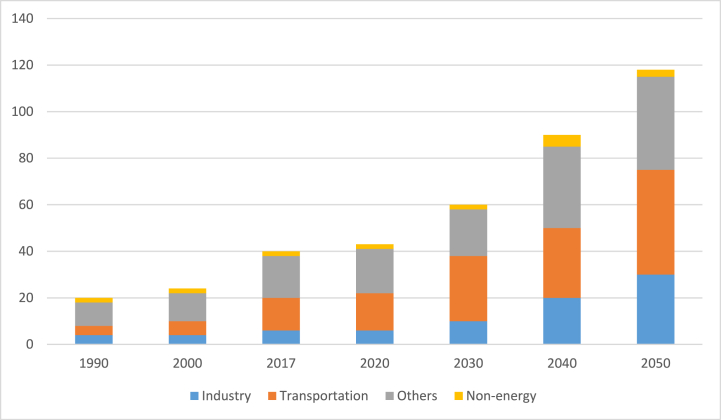
Utilization of net final energy by sectors.

**Fig. 8:**
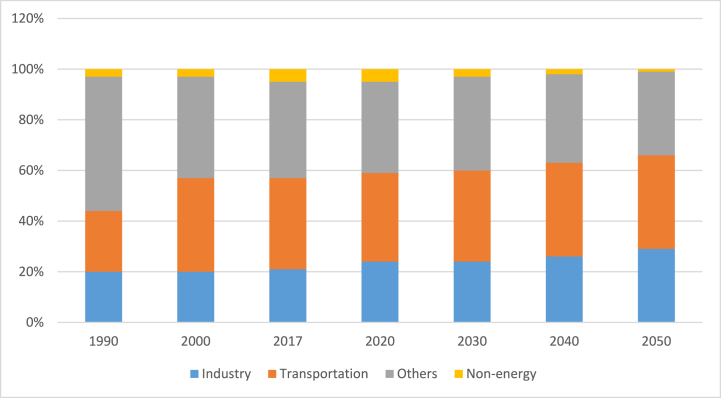
Share of net final energy consumption [59].

From 19.1 Mtoe in 1990 to 36.65 Mtoe in 2017 [3,46], the Philippines' primary utilization climbed at a rate of 2.45% per year. During this time, energy demand in the transportation sector increased by 3.45% annually, while demand in the industry increased by 2.39%. Residential, commercial, and AFF (others) had the largest part of the final energy consumption mix in 1990, at 51.6%, but decreased to 41.7% in 2017 because of a slow growth of 1.7% each year [59]. Meanwhile, under the Business-as-Usual (BAU) scenario, final energy consumption is predicted to expand at a 3.6% annual average pace from 2017 through 2050. By 2050, the remaining industries' collective requirement will account for 36.6% of total final energy utilization, notwithstanding a slower annual growth rate of 3.1%. This could be connected to the commercial sector's constant growth as services and the work environment strengthen and the government's agricultural modernization initiatives. On the other hand, transportation will continue to be the most energy-intensive sector, contributing 32.27% of total energy utilization in 2017 and growing at a 3.97% annual rate. A 4.1% annual growth in the country's industrial sector is expected as the country's economy is booming, as shown in Fig. 7, Fig. 8 [46].

**Fig. 9:**
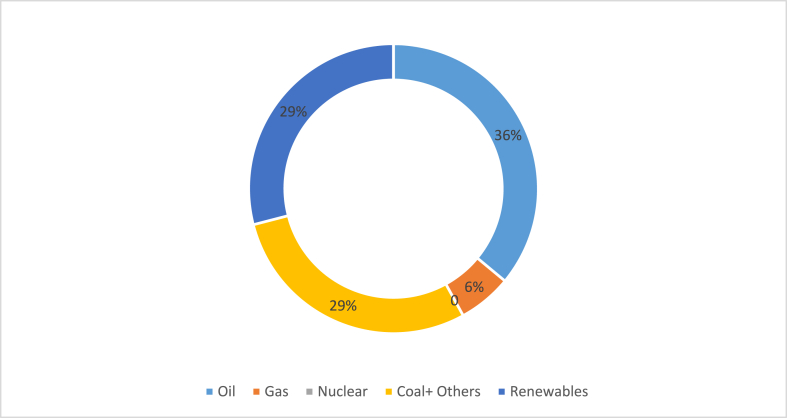
Energy mix in the Philippines in 2018 [61].

Moreover, the Philippines primarily depended on fossil fuels to generate energy. The high contributor to the national energy mix was oil at 36%, followed by coal at 29% and natural gas at 6% [60]. Next, renewable energy contributed to 29% of the nation's energy mix, with wind and solar accounting for 15%, biofuel and waste accounting for 13%, and hydro accounting for 1.3% [61], as shown in Fig. 9. Solar PV has been increasing in the Philippines for years as the government focuses on developing solar PV [62].

The government introduced the Renewable Energy Act of 2008 [63] to assist in the transition from fossil-fuel-based to RE-based electrical generation, as coal-fired and oil-based power plants generate the vast majority of electricity in the Philippines, and to make RE investments in the country more competitive. The Act established a net-metering policy framework, an incentive program administered by the Energy Regulatory Commission (ERC) that encourages customers to participate in renewable energy generation in exchange for exporting excess electricity to a distribution utility.

It is demonstrated by the fact that the Philippines' solar energy capacity has grown dramatically over the last decade [64]. From two megawatts in 2011, the figure is expected to rise to 1048 MW by 2020. The Renewable Energy Act of 2008 assists the country in increasing its renewable energy capabilities and moving away from traditional energy sources.

Any excess electricity produced by the user is immediately transmitted to the distribution network of the DU under the NEM. The DU then charges the user's electric account with a peso credit equivalent to the DU's blended production cost, minus any extra production adjustments, and excludes the credits from the bill. Customers pay P9.985 per kilowatt-hour (kWh) for power supplied by the grid [65]. The customers can also sell extra electricity generated by its solar rooftop for as little as P5.42 per kWh, as shown in the figure below. In the Philippines, NEM initiatives encourage customers to participate in renewable energy generating and help towards a low carbon emission environment [66]. In addition, the Philippine government wants to grow solar PV installations to 3 GW of utility solar by 2022. Furthermore, by the end of 2030, total solar installations are expected to reach 8.7 GW, with solar rooftops accounting for 35% of total installations. The Philippines' solar energy sector to growing even faster because of the NEM initiatives [67].

In 2020, The Energy Regulatory Commission (ERC) [68] changed its net metering guidelines to attract more individuals to engage in a scheme that permits electricity end-users to supply electricity to the Grid. The ERC announced a change to its renewable energy net-metering scheme, which allows a regular electricity user to become a "prosumer," generating power for his use and selling any excess generation to the distribution system. The Amended Net-Metering Rules stipulate that the Distribution Utilities (DUs) must complete the interconnection process within 20 days of receiving the letter of interest, provided all essential permits and licenses from various agencies are obtained. The Distribution Impact Study (DIS) charge and other relevant soft fees were also eliminated to encourage end-user engagement. The ERC has determined that conducting DIS is a regular activity of the DU to ensure the Distribution System's reliability and stability. Furthermore, the ERC's Amended Net-Metering Rules are designed to empower consumers by allowing them to generate their electricity and, as a result, pay lower electricity bills to their utility which blends renewable and cutting-edge technologies at a reasonable [69].

### 3.4 Indonesia

The state has power over Indonesia's substantial energy supplies, as specified in Article 33, paragraph (3) of the Constitution. "Earth, water, and the natural resources contained therein are governed by the state and exploited for the greatest welfare of the people," the 1945 Constitution states. In precise conditions, Article 33 paragraph (3) of the Republic of Indonesia's 1945 Constitution has three (three) crucial aspects: 1. Substance (natural resources); 2. Status (state control); and 3. Purpose (for the people's ultimate well-being). Control and utilization of natural resources are vital to the nation's survival, per the Constitution, as is the formulation of energy policy for the maximum welfare of its people.

**Fig. 10:**
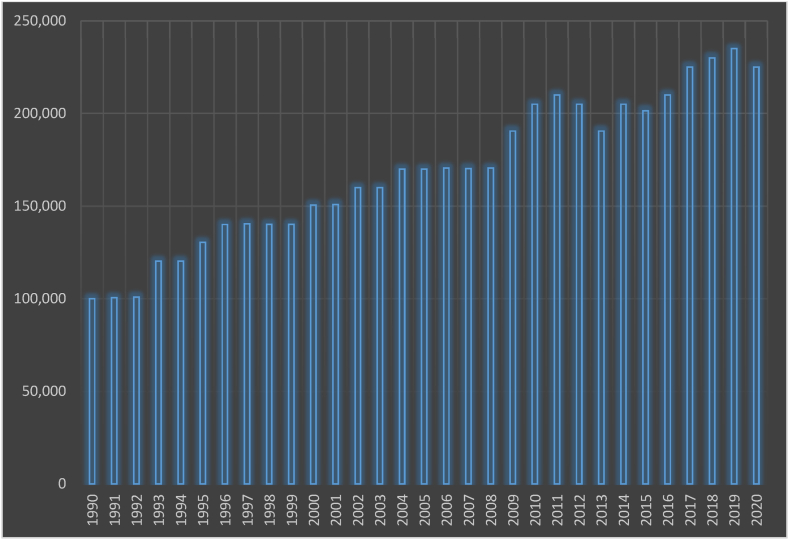
Indonesia's total energy consumption [70].

**Fig. 11:**
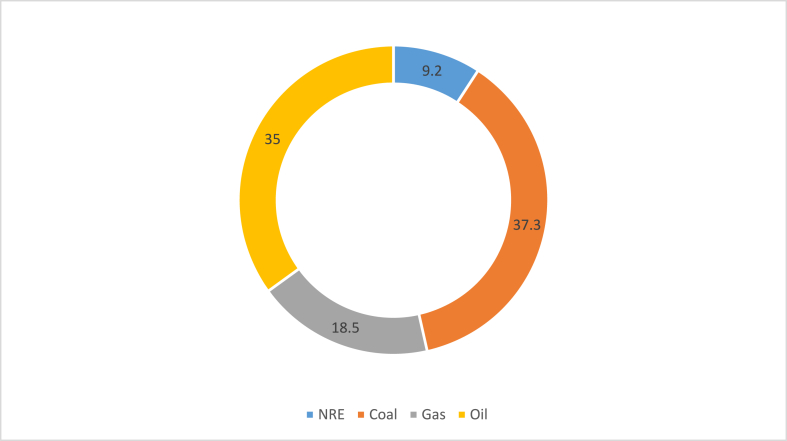
Indonesia's major source of energy in 2019 [70].

The overall consumption per capita in Indonesia is 0.8 toe, whereas the per capita energy usage is 970 kWh (2020). As indicated in the bar chart Fig. 10, total energy usage climbed by 3.09% each year from 2013 to 2019 before dropping by roughly 5% in 2020 [70]. Furthermore, Indonesia's energy usage per capita in 2020 was estimated to be around 1,0788 kWh. The government has been steadily increasing the electrification price in recent years, so it's not surprising that energy usage per capita has also increased [71]. As shown in Fig. 11, Indonesia's principal electricity generation mix in 2019 comprised 34.9% oil, 37.29% coal, 18.48% gas, 2.5% hydropower, 1.7% geothermal, 2.93% biofuel, and approximately 1.99% biogas, the solar, wind, and other renewables. Transportation accounts for 43.98% of total energy use by sector, followed by industry at 38%, households at 13.98%, and commercial at 4.5%.

According to IRENA, Indonesia currently has 148.97 billion tons of proven resources for coal, with 36.6 billion tons of potential resources; oil has 2.48 billion barrels of proven resources, with an additional 1.30 billion barrels of potential resources; and natural gas has 49.74 billion barrels of proven resources and 27.55 billion barrels of potential resources as of 2019 [[71], [72], [73]]. Moreover, the national audit office found that the nation has the highest global geothermal energy capacity of 24 GW (GW) and a hydropower capacity of more than 95 GW. The country is also rich, with more than 31.7 GW of biomass and a biogas resource of 205,000 barrels per day. The countries also demonstrate progress with renewable energy projections, with a potential of 60.6 GW for wind resources, 209 GW for solar energy, and 17.88 GW for the ocean and tidal energy [71,74]. With the fast development of renewable energy technology, the capacity might be much more significant. As a result, the government's overall renewable energy plan promotes biodiversity, ecological sustainability, and optimal solar energy harvest [74].

The Indonesian Government is planning to stimulate the development of solar PV systems on rooftops, in line with the policies it began to introduce with the issuance of Minister of Energy and Mineral Resources (MEMR) Regulation No. 49 of 2018 ("Reg 49″), to encourage Indonesia's solar PV industry, and its global commitments to reduce greenhouse gas emissions [75]. Moreover, in this report, the Residential, commercial, and industrial PV installations are permitted to export surplus power to the Grid under a net metering plan under Regulation No. 49 of 2018 ("Reg 49″). The government expects this NEM to install roughly 1 GW of PV systems in the country over the next three years, with PV system owners preserving up to 30% of their energy bills. The NEM 2018 aims to encourage PV installations with a high self-consumption rate, with just a small portion of the electricity transferred to the utility, Perusahaan Listrik Negara [76].

The government has chosen to improve the provisions again this year with MEMR Regulation No. 4 of 2020 to raise volumes and promote development [77]. Its goal is to encourage more industrial customers to install solar panels to save money on their energy costs. In October 2019, the Indonesian government changed the net metering regime. MEMR Regulation No. 26 of 2021 on Rooftop Solar Power Plants Linked to the Electricity Grid for Public Utility License Holders repealed Regulation No. 49 of 2018 ("Reg 49″). ("Reg 26″). Reg 26 became effective on August 20, 2021 [78]. The key change highlighted in Reg 26 is that it covers customers of all electricity license (IUPTLU) holders with a business area, both in PLN and non-PLN business areas (for example, in industrial estates where a third-party company is the sole electricity supplier) as Reg 49 only applies to PLN's customers. Additionally, Under Reg 49, if net energy exported from a rooftop solar PV to PLN's Grid is higher than the PLN-generated energy consumed by the customer in a single month, the difference would be credited against future invoices, provided that the credit could only be set off against PLN invoices in months falling within the same calendar quarter [75]. Under Reg 26, the time limit is extended to six months (January–June and July–December). Furthermore, PLTS construction approval can be obtained more quickly under Reg 49. According to MoMER Regulation 26/2021, just 5 working days are needed to process a PLTS construction approval, instead of the 15 working days required by MoMER Regulation 49/18 [Art. 7 (3) of MoMER 26/2021].

It is envisaged that the proposed integrated RE law and policies would bridge the gap between Indonesia's current laws and regulations and help the country meet its national renewable energy mix target. An integrated and comprehensive renewable energy regulation has proven to be able to optimize the renewable energy development process in several other countries. For example, countries in the Asia Pacific region, including Australia (since 2000), Japan (2003), China (2006), Sri Lanka (2007), Mongolia (2007), Philippines (2008), South Korea (2010), Pakistan (2010), and Malaysia (2011). The proposed New Renewable Energy Law (RUU) in Indonesia has been included in the DPR's 2020–2024 priority National Legislative Program (PROLEGNAS). It is hoped that all these energy policies can become an umbrella regulation to support the successful implementation of national energy policies so that Indonesia can achieve the target of 23% of the renewable energy mix in 2025. The Indonesian government has declared its participation in the Sustainable Energy Demand Project (CEDI), a US government initiative to encourage investment in the clean energy industry. Indonesia supports the international community in implementing climate change mitigation and improving the green economy (green economy). According to the Clean Energy Demand Initiative, the president's directive is a real international assistance initiative to boost steps to achieve the NDC target by 2030 and becoming a Net Zero Emissions by 2060," stated Minister of Energy and Mineral Resources Arifin Tasrif at the launch of CEDI at the COP26 event at Pavilion US, Glasgow, the United Kingdom on Thursday (4/11) local time.

President Joko Widodo has declared that Indonesia must be encouraged and supported in its energy transformation towards new and renewable energy (EBT). "Indonesia needs to enhance its green economy, green technology, and green products in order to compete in the global market," stated Arifin. In addition, a Super Grid will be constructed beginning in 2025 in order to provide local communities throughout Indonesia with energy access. It is also being backed by a change in regulation surrounding the use of an existing shared network (power wheeling) to allow the direct transmission of electricity from EBT sources to the company's operating facilities in an effort to advance the Green Grid's advancement further.

For the record, Indonesia has set a 2025 goal of 23% NRE in the primary energy mix, a reduction in emissions of 29–41% based on the Nationally Determined Contribution (NDC) target for 2030, and a goal of Net Zero Emission (NZE) by 2060 or earlier with international help (target). Meanwhile, as the largest economy in Southeast Asia, it is understandable that Indonesia has substantial growth in energy demand. National Energy Council estimates that energy consumption will rise at a pace of between 4.3% and 5.0% by 2050. With current trends, energy demand will double by 2030 [79].

The Minister of Energy and Mineral Resources emphasized that this financial support is an essential element alongside the preparation of the regulatory framework that the government has prepared in the form of the Electric Power Supply Business Plan (RUPTL 2021–2030), the Roadmap towards Net Zero Emission 2060, as well as regulations related to the carbon economy Overall, the Indonesian government's implementation removal of the discount on exported energy, the extension of the set-off period, the introduction of quicker licensing processes, and integrated renewable energy law and policies will generate significant interest among customers seeking to develop rooftop solar PV projects under NEM scheme [80].

### 3.5 Malaysia

The government has entrusted Suruhanjaya Tenaga as a national utility company that fully supports government initiatives related to NEM because the capacity of solar PV installations in the electricity sector can be increased, allowing users to implement solar PV systems for self-consumption while also receiving excess energy channeled for the NEM scheme by the approved consumer [81].

Malaysia's Ministry of Energy and Natural Resources (KeTSA) adopted this environmentally friendly initiative on October 6, 2016, governed by the Energy Commission and administered by the Sustainable Energy Development Authority (SEDA) Malaysia (SEDA). As part of Malaysia's Eleventh Plan, this program encourages the use of Renewable Energy (RE) (RMK-11) [82,83]. The purpose of the FIT system was to assist and develop the RE industry. Nevertheless, the FIT plan is challenging to sustain since other electricity customers are forced to contribute to the system through a renewable energy fund levy that is included in their monthly bill [84,85]. It is collected by charging a 1.7% surcharge on the power consumers use. TNB's job is confined to that of a government fund collector. As a result, In 2016, NEM was launched to address these challenges [86].

Two years after its release and widespread implementation, NEM 2016 still hasn't helped RE reach its expansion goals. Because each kWh unit of imported energy would cost RM0.31, it isn't financially attractive to pay for it that way. According to Table 1, residents of Malaysia are charged according to Tariff A (domestic tariff) [85,86]. After 200 kWh, customers will pay 21.8 cents per kWh and 33.4 cents per kWh for each additional 100 kWh. If they use more electricity, their electricity bill will go up. After the 301th kWh, they will be charged 51.6 cents per kWh, which is much higher than the displacement cost for NEM payment if their monthly use exceeds 300 kWh. High-energy consumption families may not profit from the NEM system. Small customers (less than 200 kWh per month) may not benefit from PV because they already pay a cheap electricity cost [87]. According to the World Energy-BP Statistical Report, the total installed solar energy power in 2021 is 700GW, which has been raised up to 94% from 2010. Malaysia is not lagging in this current development of solar energy with successful solar initiatives but is moving forward and becoming one of the leading countries in the Southeast Asian region.

**Table 1:** Tariff (domestic tariff) [88].

Tariff Category (kWh)	Unit Price (cent/kWh)
1–200	21.8
201–300	33.4
301–600	51.6
601–900	54.6
>900	57.1

#### 3.5.1 NEM 2016

Solar energy is a permissible technology under NEM 2016. The NEM mechanism allows users to generate electricity from renewable energy resources such as solar, where consumers first use that energy for self-consumption. NEM uses a bidirectional meter. The generating capacity limit is 1 MW. If there is excess energy, it will be sold to the electricity suppliers company such as TNB or SESB. The validity period termination of NEM is entirely based on the NEM or local electricity supply contracts. Clients in the residential, commercial, and industrial sectors can use it. Single-phase residential capacity is 12 kW, while three-phase residential ability is 72 kW [9]. Up to 1 MW of electricity can be used in commercial and industrial applications. Excessive production will be allocated at displacement cost to the following billing period. The displacement cost is the average cost of producing and providing a 1-kW hour of power from non-renewable resources along the supply line up to the point of interconnection with the RE installation. As shown in Table 2, the net power consumption or credit can be carried forward for 24 months before any remaining credits are forfeited. Table 2 [89] shows the full structure of NEM 2016.

**Table 2:** Structure of NEM 2016 [89].

Allowable Technology	Allowable Customer	Allowable Capacity	Net Excess Generation (NEG)
Solar 1. Building's roof 2. Parking space or similar structure [99]	Residential, Commercial, Industrial [87]	Residential-12kWp (single phase) 72kWp (3 phase) Commercial and industrial- 1MWp or 75% of maximum demand or 60% of fuse rating or 60% of current transformer	Any surplus will be credited at a displaced cost in the next billing month. The maximum term of rollover is 24 months. After 24 months, any remaining credit will be forfeited.

The Net Energy Metering (NEM) applicant must be a registered consumer of the Distribution Licensee (DL) in the Peninsula, Sabah, and Labuan during the application period [90]. The only way to connect the NEM scheme to the Distribution Licensee Network is through an indirect connection. Before a NEM application is approved, the applicant must conduct a NEM Assessment Study to establish the technical feasibility of connecting the proposed installation to the Distribution Licensee's electricity distribution network. The study's findings aid the NEM applicant in determining the project's cost feasibility and the Distribution Licensee in preparing the technical requirements for interconnection. The credit to the customer in the event of NEM must be calculated using the current gazette displaced cost for the relevant supply voltage level at the common coupling point. The gross bill of electricity will be calculated using the formula below.

Net Billing=(Energy Consumed from DL (kWh) × Gazetted tariff)-(Energy Exported to DI × Displaced Cost.

#### 3.5.2 NEM 2019

Only a few modifications have been made to NEM 2019 compared to NEM 2016. One-to-one offsets will be paid to national company Tenaga Nasional Berhad (TNB) for surplus solar PV generated when the building has been completed. All TNB customers, whether residential, commercial, industrial, or agricultural, are eligible to participate in the program. When it comes to selling and purchasing power, the NEM 2.0 mechanism provides NEM members with the same tariffs for both transactions. Instead of being charged at "Displaced Cost" as before, every kWh exported to the Grid will equal the kWh used from the Grid. Tariff A (domestic tariff) will then be used to credit the surplus generation in the subsequent billing month [88]. Customers can keep any credit earned if the solar rooftop generates more energy than the premise consumes. For up to 24 months, the kWh credit can be used. Other than that, the NEM 2016 system is the same as NEM 2016. Table 3 shows the specifics.

**Table 3:** Structure of NEM 2019 [92].

Allowable Technology	Allowable Customer	Allowable Capacity	Net Excess Generation (NEG)
Solar 1. Rooftop of building 2. Garage, Car Park, or similar building	Residential, Commercial, Industrial. Agriculture	Residential-12kWp (single phase) 72kWp (3 phase) Commercial and industrial- 1MWp or 75% of maximum demand or 60% of fuse rating or 60% of current transformer	Any excess will be credited at the retail rate in the next billing cycle. The maximum term of rollover is 24 months. After 24 months, any remaining credit will be forfeited.

As a result of this new approach, more consumers are likely to implement rooftop solar systems and register with NEM. Limits for commercial and industrial customers are 75% of the maximum demand (MD), whereas low voltage customers are limited to 60% of their current transformer rating or the fuse's capacity. Three-phase systems can produce 72 kWp, but single-phase systems can only produce 12 kWp. 500 MW of the 2019 NEM quota has been allocated to industrial and commercial construction, with 450 MW allocated to the Ministry of Science, Technology, Environment, and Climate Change. According to SEDA, if all of Peninsular Malaysia's rooftops are fitted with solar panels, Malaysia will be able to generate more than enough electricity to meet its current demand under the NEM scheme [91].

#### 3.5.3 NEM 2021

This initiative, dubbed "Net Energy Metering 3.0" ("NEM 3.0″), is set to go live on December 30, 2020, according to the Ministry of Energy and Natural Resources (or "KETSA") [93,94].

After the previous "NEM 2.0″ allowance of 500 MW was used up on November 29, 2020, this notification was made. From 2021 to 2023, a quota of 500MW would be set aside for the NEM 3.0, a government program to boost Malaysian solar energy investment. NEM 3.0 will offer three key new measures to enhance solar energy usage, as mentioned below.

##### 3.5.3.1 NEM Rakyat (domestic consumers)

Successful candidates will get a 10-year contract under the NEM Rakyat program from February 1, 2021 [95]. The program runs through the end of 2023 or when the quota is exhausted (whichever happens first). Until all available quotas are used up, the NEM Rakyat program adheres to the 1-to-1 offset principle. For Tenaga Nasional Berhad domestic account customers or around 40,000 to 100,000 families in Peninsular Malaysia, the program offers discounts on their power bills. Ten years of offset rates will be provided to customers, after which they will see a reduction in their power prices due to self-consumption. A rise in the number of people working from home might lead to a surge in household power usage, given that NEM 3.0's expected savings could encourage more people to do so.

##### 3.5.3.2 NEM GoMEn (Government Ministries and Entities)

NEM GoMEn, the second project under NEM 3.0, is a scheme that reduces electricity expenses for government buildings and workplaces. From February 1, 2021, to December 31, 2023, SEDA will accept applications for quota allocation under Program GoMen on a first-come, first-served basis. An application fee of RM10.00 would be levied for each kW sought. According to KETSA, roughly one hundred (100) government organizations in Peninsular Malaysia will save a total of RM6 million per month on electricity expenses due to this scheme [94].

A property owner or occupier must be a TNB customer to be qualified for quota allotment under the NEM Rakyat or NEM GoMEN programs, respectively [97]. This requirement was already in place when NEM 2.0 was released. However, under the NEM 3.0 Guidelines, a new restriction states that anyone who has established a solar PV installation under NEM 2.0 is ineligible to participate in NEM Rakyat. A solar photovoltaic system under NEM Rakyat or NEM GoMEn must be in the form of photovoltaic panels erected on the rooftop of the building within the premise provided with power by TNB, according to the NEM 3.0 Guidelines. Installations in car parks and ground-mounted installations, unlike NEM 2.0, will not be permitted or evaluated. The following is the highest capability of an installation under NEM Rakyat and NEM GoMEn shown in Table 4.

**Table 4:** Structure of NEM 2021 [94].

NEM 3.0 Initiatives	Capacity
NEM Rakyat	i) 4 kW for a single-phase system; and ii) 10 kW for a three-phase system
NEM GoMEn	Maximum capacity of 1,000 kW. The following requirements must be met: i) medium-voltage customers must not surpass 75% of their maximum consumption ii) Low-voltage customers must not exceed 60% of the fuse rating (for direct meters) or 60% of the current transformer rating of the monitoring current transformers [94]. The NEM 3.0 rules define maximum demand as twice the highest amount of kilowatt-hours used in any consecutive 30 min in a month.

The NEM 2.0 '1 to 1 offset' idea ('Offset') is kept and applied to both NEM Rakyat and NEM GoMEn. Which implies to any power generated by the consumer's installation but not used owing to operational restrictions or changes in load needs can be exported to the Grid. After that, the consumer will get credits for the energy that was exported, which they may use for a portion of their TNB-supplied power payment for the corresponding billing period. It is possible to roll over net credits for twelve months every calendar year. The offset will only be available for the first 10 years after the consumer's TNB contract begins, after which the consumer's power usage will be transferred to self-consumption.

##### 3.5.3.3 Program Nova

The program NOVA was developed as part of NEM 3.0 and is intended for commercial and industrial customers that the COVID-19 outbreak has impacted. Proposals for quota allotment under Program NOVA may be submitted to SEDA from April 1, 2021, through December 31, 2023, or until the quota is fully assigned. As of this writing, the Energy Commission (EC) has not yet established guidelines for Program NOVA. The present state of knowledge on the elements of Program NOVA is as follows:

i. A quota of 300MW has been assigned.
ii. Instead of the existing tariff, the offset will be based on the System Marginal Price ('SMP') rate. The subsequent month's power bill will turn this into credits.
iii. For up to three power billing accounts held by the same person, virtual aggregation of excess electricity generated by rooftop solar installations is possible.
iv. Whether the user chooses Offset just (1MWac) or Offset with virtual aggregation, the capacity limit will be established (5MWac).

KESAS has said that the program's goal is for entrepreneurs, small enterprises, and houses of worship to alleviate their power bills and operational expenses. NEM Rakyat Program, NEM GoMEn (Government Ministries and Entities) Program, and NOVA (Net Offset Virtual Aggregation) Program are the three main initiatives that seek to mitigate carbon emissions and explore alternative approaches for green energy sources and sustainable production. Table 5 presents the overview of these three initiatives.

**Table 5:** Overview of NEM 3.0 mechanism [94].

Subject	NEM Rakyat (Domestic)	NEM GoMEn (Government Building)	NOVA (Commercial & Industrial)
Quota Offered (MW)	100	100	300
Mechanism (roll over)	NEM 1:1 (12 months)	NEM 1:1 (12 months)	SELCO + (1 month)
Commencement Date	February 1, 2021	February 1, 2021	April 1, 2021
Offer Duration	3 years		
Offset Rate	Current Tariff	Current Tariff	System Marginal Price
Setting After 10 years	Self-Consumption (SELCO)		
Installation Capacity Limit	Single Phase: 4 kWac Three Phase: 10 kWac	1MWac/1 account	Net Offset: 1MWac Net Offset + Virtual Aggregation: 5MWac
Eligibility	Domestic Account Holder	Government buildings	Non-domestic Account Holder

### 3.6 Net-metering growth towards low carbon emissions

**Fig. 12:**
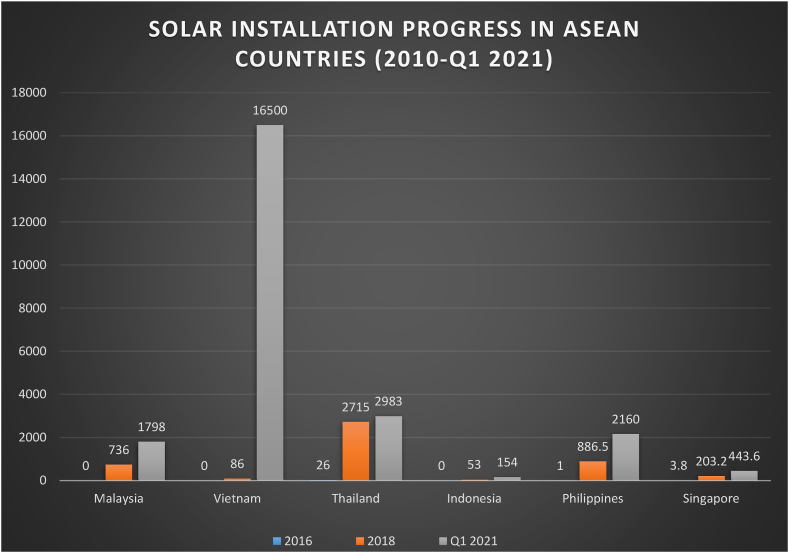
Solar installation progress in ASEAN countries [91,96].

An overview of net metering regulations, tariff rates at which surplus energy is fed into the Grid, and subsequent goals and present power output capacity from renewable energy sources are given in Table 6. Across all facts, the region's renewable energy (RE) implementation has accelerated, especially in solar installation under the net metering policy. Between 2010 and 2019, the total generating capacity from solar installation shot up from 1 MW to 9 GW, according to International Renewable Energy Agency (IRENA) statistics. As illustrated in Fig. 12, by the end of 2021, Vietnam, Thailand, and Malaysia dominated the Asian race with renewable energy capacity factors of 16.5 GW, 2.9 GW, and 1.8 GW, correspondingly.

**Table 6:** Net-metering development in Southeast Asian Countries.

Ref.	Country	Eligible Sectors	RE Capacity Cap	RE Target	Tariffs for Surplus Energy fed into the Grid
[37,43] [97,98]	Thailand	commercial, industrial, residential	Up to 1MWp	Renewable energy will make up 22% of overall power output and 32% of energy demand by 2036.	THB 9.0–12.0 per kWh for net metering; THB 3.0–4.5 cents per kWh for net billing [96]
[67,98] [99,100]	Philippines	Residential, commercial, industrial, institutional	Up to 100kWp	100% by 2050 (20GW by 2040)	Tariff for net metering (fixed tariff): 10.89 Philippine peso cents per kWh (excess energy is credited to the following month's electricity bill) [96]
[83,101,102]	Indonesia	Commercial, residential, industrial	The annual energy usage of qualified users should not exceed the generation from DG systems.	26% of installed capacity by 2025	Tariff for net metering: 11–13 Indonesian rupiah cents per kWh (net metering is not on a 1:1 basis; the excess energy is fed into the Grid at 65% of the applicable tariff) [96]
[63,103,104]	Vietnam	Residential sector	No capacity limit is imposed	By 2020, 2030, and 2050, renewable energy will account for 7%, 10%, and 100% of total electricity generation, respectively.	Tariffs for net metering range from 6.9 to 12.99 Vietnamese dong cents per kWh.
[97,104,105]	Malaysia	Residential, commercial, industrial, agriculture	Residential 1-phase:12kWp, 3-phase:72kWp. Commercial: up to 1MWp or 75% of their maximum demand (whichever is lesser). Industrial: 60% of the fuse rating	By 2020 and 2030, the share of renewable energy will be 10% and 22%, respectively.	Net metering tariff: 21.80–57.10 MYR cents per kWh (every supplied energy unit into the Grid is compensated by a purchased energy unit from the Grid). The maximum period of rollover is 24 months) [96]

Moreover, the net metering legislation's effectiveness reflects Southeast Asian governments' efforts to promote renewable energy initiatives as part of sustainable biodiversity protection [91]. Almost all Southeast Asian countries have made net-zero emission promises, accounting for nearly all of the region's carbon output. Sustaining the pace of this rising commitment and capitalizing on emerging decarbonization alternatives is critical, as the path to net zero in the region is limited and brief. The Conference of the Parties (COP) of the United Nations Framework Convention on Climate Change (UNFCCC) in 2021 was possibly significant in highlighting Southeast Asia's increased climate ambition [96]. By the end of COP26, eight out of ten ASEAN Member States (AMS) had vowed to achieve emissions reductions, with Vietnam and Thailand expressing their intentions at the conference after Lao PDR, Indonesia, and Malaysia announcements before the conference [96]. By continuing this path and with the success of the net metering policy, it's confident that Southeast Asian countries could reach the 2050 target on carbon capture methods would need to remove at least 200 million tons of emissions.

## 4 Material and method

**Fig. 13:**
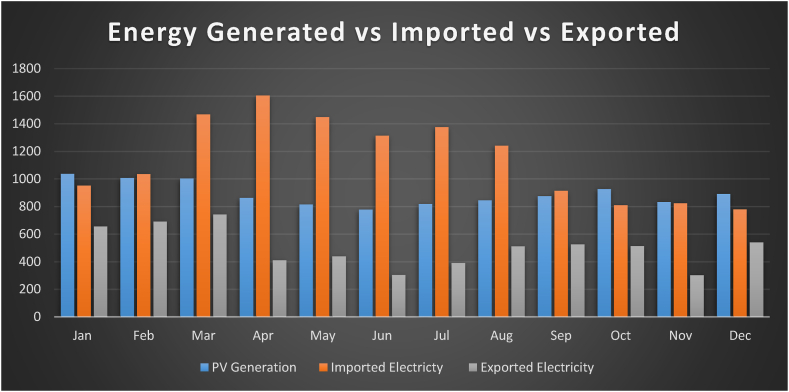
PV generation, energy imported, and energy exported totaled during the year 2020.

Implementation of Net Energy Metering are advancing and widespread, this case study that into account in this section to evaluating the financial benefits to consumers. The Block F1 building in UTHM, Johor Malaysia, is the subject of interest in this study for a thorough analysis of NEM2.0 policy achievement. The load profile of the site, specifically the PV generation ($E_{pv}$), energy imported ($E_{imported}$), and exported ($E_{exported}$). were collected for this analysis. Fig. 13 displays the monthly statistics for these variables for the year 2020. Table 7 summarizes the PV system's characteristics.

**Table 7:** Summarizes the solar PV system's characteristics.

Parameter	Values
Total PV capacity	8.28 kWp
Total of PV modules	24 units
Roof covered area	36m2
Inverter Capacity	1 unit 8 kW
Project lifetime	20 years
Capital Cost	RM28630.00

Equations 1, 2) can be used to determine the total energy used by the load ($E_{load}$) and the amount of PV energy that was self-consumed ($E_{self\;consumed\;PV}$) from these data.

1 $$E_{self\;consumed\;PV}=E_{pv}-E_{exported}$$

2 $$E_{load}=E_{self\;consumed\;PV}+E_{imported}$$

From $E_{load}$, a conventional calculation using the tariff shown in Table 1 can be used to estimate the power cost from before the PV system was built (before NEM2.0). The monthly savings can be obtained from the collected data based on equation (3).

3 $$Savings={Bills}_{before\;NEM2.0}-{Bills}_{after\;NEM2.0}$$

Then, using the following equation (4), a simple payback period (PB) may be calculated [9] that illustrates the quickest time for the whole initial investment to equal the cumulative economic savings.

4 $$PB=\frac{Capital\;Cost}{saving}$$

The savings in equation (3) are not net savings, but rather monthly savings from lower electricity costs. By treating all costs incurred over the course of the project as a net present cost (NPC), it is possible to compute savings in a more precise manner (NPC). The NPC value takes into account all expenses incurred throughout the course of the project, including capital expenses, maintenance costs replacement expenses, salvage costs, and grid sales revenue. The following equation (5) can be used to determine the NPC while discarding the annual real discount rate.

5 NPC(RM)=Totalexpenditure(RM)+Totalrevenue(RM)

The cost of energy (COE) in equation (6), which displays how much the consumer must pay per kWh, is another crucial factor.

6 $$COE\;\left(\frac{RM}{kWh}\right)=\frac{Annualized\;total\;cost\;\left(RM\right)}{total\;consumption\;\left(kWh\right)}$$

## 5 Results and discussion

The economic study performed prior to installing the PV system is described in this section. The correct method for calculating the export bill is then given using the actual NEM 2.0 electricity bill as a basis. The performance of NEM 2.0 is then discussed using data that has been gathered.

### 5.1 Grid connected system

The economic characteristics employing a grid-connected system were first examined before considering the influence of the NEM system., $E_{load}$ was computed from equation (2) to determine the annual energy usage, which was 18, 416 kWh. The yearly energy bill prior to the implementation of NEM was then computed using Table 1 and revealed to be RM 9098/year. The annual bill before NEM was multiplied by 20 years to get the NPC for this system, ignoring the upfront costs borne by the consumer during the construction of the grid connection. In the event that the indicator is remains same for 20 years, the NPC prior to NEM would be RM 181960.00. The COE before NEM was determined using the annual bill and energy usage, and it revealed a value of 0.494 RM/kWh.

### 5.2 Export & import electricity bill calculation

This section explains the formula used to determine the NEM 2.0 electricity bill. $E_{imported}$ and $E_{exported}$ are factors. First, using Table 1, the import bill was computed similarly to a regular electricity bill. The government calculates the export bill in reverse order beginning with the higher block tariff, subject to the maximum block tariff used in the import bill, in order to incentivize RE investors to use the NEM programme.

Here, two samples based on the actual bill are provided. In Tables 8 and 9, the electricity bills for the months of April and November are shown as examples. In April, the maximum amount of electricity was imported (1,602.93 kWh) but $E_{exported}$ was low, while in November, $E_{imported}$ was 823 kWh and $E_{exported}$ was 301.728 kWh.

**Table 8:** Imported and exported bill calculations for April 2020.

Imported Bill			Exported Bill		
Tariff	Usage	Amount	Tariff	Usage	Amount
0.218	200	43.6	0.571	409.48	233.81
0.334	100	33.4	0.546	0	0
0.516	300	154.80	0.516	0	0.00
0.546	300	163.80	0.334	0	0.00
0.571	702.93	401.37	0.218	0	0.00
Total	1602.93 kWh	RM 769.97	Total	409.48 kWh	RM233.81

**Table 9:** Imported and exported bill calculations for November 2020.

Import Bill			Export Bill		
Tariff	Usage	Amount	Tariff	Usage	Amount
0.218	200	43.60	0.546	223	121.76
0.334	100	33.40	0.516	78.73	40.625
0.516	300	154.80	0.334	0	0
0.546	223	121.76	0.218	0	0
Total	823 kWh	RM 353.55	Total	301.73 kWh	RM 162.3

### 5.3 Before NEM 2.0 VS after NEM 2.0

**Fig. 14:**
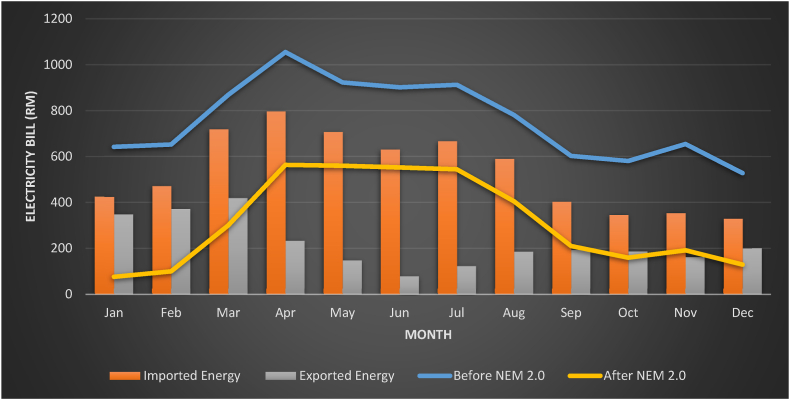
Energy bills for imported and exported as well as before and after NEM 2.0.

Bill before NEM 2.0, which was heavily reliant on load usage, and Bill after NEM 2.0, which was computed in the preceding section, are shown in Fig. 14 below. The figure describe that January and February recorded lowest Bill after NEM 2.0 as more electricity exported to Distribution Licensee. Apparently, April to Jun recorded higher bill after NEM 2.0 due to high electricity supplied by Distribution Licensee. The tabulation and summary of the annual data collection are provided in Table 10.

**Table 10:** Annual data collection for the case study.

Parameters	Values
Annual $E_{pv}$	10678 kWh
Annual $E_{imported}$	13,750 kWh
Annual $E_{exported}$	6011 kWh
Annual $E_{self\;consumption}$	4667 kWh
Annual $E_{load}$	18,617 kWh
Annual ${Bill}_{before\;NEM2.0}$	RM 9098
Annual imported bill	RM 6441
Annual exported bill	RM 2263
Annual ${Bill}_{after\;NEM2.0}$	RM 3788
Annual saving bill	RM 5310

The table makes the benefit of NEM 2.0 abundantly evident, with the annual ${Bill}_{before\;NEM2.0}$ lowered to RM 3788 for the ${Bill}_{after\;NEM2.0}$ , an about 58% savings. This decrease is a result of NEM 2.0's annual savings, which were computed using eq (5). The PV investor's simple payback term may also be calculated using eq (6), which in this case, the value is 5 years 5 months. . NPC was calculated Considering that an inverter is changed every 15 years, and all the data collection is maintained every year, then the NPC afterNEM for 20 years can be calculated and the value would be RM 80,263. The COE after NEM2.0 is likewise decreased to RM 0.203 per kWh, demonstrating how connecting a PV system with the grid lowers the cost of electricity.

## 6 Limitations and key challenges of NEM implementation

Accelerating clean energy transitions globally will put further strain on solar PV supply chains, with demand increasing massively in a NetZero pathway. There are many whys and wherefores to this challenge of implementing large-scale applications of NEM policy. The limitations of using the NEM policy in the solar energy sector in real-world applications are listed below.

I. Availability of Photovoltaic Power: The utility system that sufficiently utilises the amount of solar energy for electricity generation must effectively manage intermittency to maintain the stability and dependability of the Grid. Unlike a traditional electricity supply system, which generates a consistent output, solar PV's output is changeable and dependent on the weather. As a result, the solar energy output is greatly influenced by the surroundings and the weather, such as the quantity of sunshine, cloud cover, and shadow. Thus, an unbalance between supply, and demand may come from this.
II. PV Installation: The solar array's performance may vary as it gets tested in STC conditions, with radiation of 1 kW/m2, a cell temperature of 25 °C, and no wind but installed at weather variated environments (No STC condition). Everyone yearned for a PV generation system in their home. The public's favorable reception to solar panels allowed other producers and pioneers in the field to emerge. Unfortunately, the high acquisition costs and unreliable technology during its peak led many to become disinterested in solar technologies.
III. Location of Resources: Most RE facilities that connect to the Grid require a spacious area. Location is typically a factor in RE sources, which customers may find off-putting. Firstly, not all places have access to certain RE sources. Secondly, the distance between the RE source and the Grid requires high cost and efficiency. Additionally, as renewable energy sources are climatically and geographically dependent, a single kind of energy production may not be suitable for a given area.
IV. Poor Awareness among the Public: Even though this subject is progressing, there is a dearth of knowledge and understanding of its advantages and the necessity of RE. For the adoption of renewable energies, capital allowances and investment opportunities have been made available. Governmental organizations should help and direct applicants and prospective recipients on how to submit applications for renewable energy subsidies.
V. Cost Issue: Unafforrable cost of installing solar panels as solar analysts suggest that a standard residential installation could vary from 4 kW to 12 kW, with costs ranging from between RM16,000 to RM23,000 for a basic system of 3 kW.
VI. Resource Location: Most renewable energy plants that share their energy with the Grid require large space areas. In most cases, renewable energy sources are dictated by location, which can be off-putting to users. Firstly, some renewable energy sources are not available in different regions. Secondly, the distance between the RE source and the Grid is a major aspect of cost and efficiency. In addition, RE sources depend on the weather, climate, and geographical location, meaning that one type of energy generation is not appropriate for the region.
VII. Demand Side Management (DSM) pricing structure: A suitable electricity price mechanism to influence the integration level of demand side management programs. Moreover, optimal coordination of DSM programs to improve the reliability of the distribution system incorporated with highly penetrated RE sources.
VIII. Solar Modules prices: At the present price level, solar modules account for about 65% of the overall cost of setting up solar projects in Malaysia. Domestic production only makes up 20% of the annual requirement, so heavy reliance on imports from China exposes the country's ambitious renewable energy plan to geopolitical risks. Prices of monocrystalline modules have increased by more than 40% from $0.27 to $0.28 per watt over the last 18 months. In addition, the increase in solar PV system prices and the imposition of basic customs duty on imported cells and modules is leading to cost pressure.
IX. Battery Storage: RE technology for producing power and delivering it to millions of different sites is adequately developed and compelling. However, not sufficient resources to store energy to balance the variations between high-generation periods (when the sun is shining) and high-demand periods. Creating adequate large-capacity power storage still needs deep fundamental research.

## 7 Conclusion and future recommendations

The transition to sustainable and clean energy technologies has become crucial in light of mounting concerns about traditional energy resource shortages and climate change. On the other hand, RE with NEM continues to confront and reduce considerable obstacles to mainstream adoption. One of the possible motivations for increasing local distributed energy resources, particularly solar PV, is net-metering. The present situation of net-metering policies, as well as RE penetration and the future ambitions of Southeast Asian nations, were examined in this article. The existing research on NEM policies in Southeast Asian nations is also discussed. The findings demonstrated that NEM 2.0 offers consumers more advantages over grid-connected systems. A government commitment to ambitious core climate policy frameworks that is consistent and successful is a crucial driver of low-carbon innovation. The establishment of new businesses, the transformation or phase-out of conventional fossil fuels, the rise of new emerging technologies and innovations, and the development of proper support guidelines for innovations to be widely adopted with the removal of all barriers are all examples of innovation for the low-carbon transition enabled by the net metering policy. This evaluation is expected to be valuable to policymakers, energy-producing firms, research groups, and the Malaysian government.

Furthermore, the Bandar Seri Begawan Joint Declaration announced at the 39th ASEAN Ministerial Meeting on Energy (AMEM), held on September 15, 2021, is the region's country's commitment to strengthening energy security and intensifying energy transition initiatives toward low carbon. The Ministry of KETSA, informed and showed the commitment to the ASEAN Energy Minister in the form of funding, investment, and technical assistance from ASEAN Dialogue Partners. Moreover, international organizations and others to ensure the successful implementation of the ASEAN Action Plan on Energy Cooperation (APAEC). Further, ASEAN energy ministers agreed to set a goal of 24% renewable energy in total electricity generation and 36% renewable energy in primary energy supply by 2025, where another 30GW–45GW of renewable energy capacity would need to be built by 2025. Malaysia expressed its commitment to successfully implementing the Sub-Sector Network work plan, particularly under APAEC Phase II, to achieve its goals. In this sense, Malaysia shares plans for post-COVID-19 economic recovery by implementing RE projects that increase employment and open up green businesses by 2025, where the current statistic shows that with a 33% share, Vietnam leads the way in terms of sustainable transformation, followed by Thailand 16.6%, Indonesia (13.3%), Malaysia (11%), and the Philippines (11%).

Following are the recommendations for implementing RE developments in ASEAN counties:

• Formulation of government policies to take further initiative and develop ways to build sustainable energy policies and plans. To thrive in the green technology business, the necessary support mechanisms must be in place to generate a market, motivation, and interest in developing and applying renewable energy technologies.
• Efforts to lower carbon emissions can accelerate rapidly with ongoing NEM policies and public support, as poor promotion of RE and lack of introduction indicates the real struggles of ASEAN countries in aiming for a cleaner environment by avoiding CO_2_ emission. By continuing this path and with the success of NEM, it's confident that ASEAN countries could reach the 2025 target of reducing their carbon footprint.
• To extract the maximum value from climate change finance alternatives, it is necessary to develop focused, coherent, and comprehensive recommendations for renewable energy to subsidize global climate change policies.
• Implementation of Demand Side Management (DSM) with NEM arrangements to incentivize small-scale distributed RE generations.
• Simulation of tariff structure of the time of use (TOF) to facilitate the DSM because of the extensive integration plan of RE to 30–40% of the installed capacity for ASEAN countries is still an open topic.

## Author contribution statement

All authors listed have significantly contributed to the development and the writing of this article.

## Funding statement

MR Logeswaran Govindarajan was supported by Research Management Centre (RMC), Universiti Tun Hussein Onn Malaysia [RE-GG (vot No. 083.)].

## Data availability statement

No data was used for the research described in the article.

## Declaration of interest’s statement

The authors declare no conflict of interest.
